# The Impact of BRCA1- and BRCA2 Mutations on Ovarian Reserve Status

**DOI:** 10.1007/s43032-022-00997-w

**Published:** 2022-06-15

**Authors:** Drechsel Katja C.E, van Tilborg Theodora C., Eijkemans Marinus J.C., Lentjes Eef G.W.M., Homminga Irene, Goddijn Mariette, van Golde Ron J.T., Verpoest Willem, Lichtenbelt Klaske D., Broekmans Frank J.M., Bos Anna M.E.

**Affiliations:** 1 Department of Reproductive Medicine, University Medical Centre Utrecht, Utrecht University, Heidelberglaan 100, PO Box 85500, 3508 GA Utrecht, The Netherlands; 2 Julius Centre for Health Sciences and Primary Care, University Medical Centre Utrecht, Utrecht University, Heidelberglaan 100, PO Box 85500, 3508 GA Utrecht, The Netherlands; 3 Central Diagnostic Laboratory (CDL), University Medical Centre Utrecht, Utrecht University, Heidelberglaan 100, PO Box 85500, 3508 GA Utrecht, The Netherlands; 4 Department of Obstetrics and Gynaecology, Section Reproductive Medicine, University of Groningen, University Medical Centre Groningen, Hanzeplein 1, 9700 RB Groningen, The Netherlands; 5 Department of Obstetrics and Gynaecology, Centre for Reproductive Medicine Amsterdam UMC, University of Amsterdam, Meibergdreef 9, AZ 1105 Amsterdam, The Netherlands; 6 Department of Obstetrics and Gynaecology, Maastricht University Medical Centre, P.O. Box 5800, 6202 AZ Maastricht, The Netherlands; 7 GROW - School for Oncology and Developmental Biology, Maastricht University, P.O. Box 616, 6200 MD Maastricht, The Netherlands; 8 Centre for Reproductive Medicine, Universitair Ziekenhuis Brussel, Laarbeeklaan 101, 1090 Brussels, Belgium; 9 Department of Genetics, University Medical Centre Utrecht, Heidelberglaan 100, 3508 GA Utrecht, The Netherlands

**Keywords:** BRCA gene mutations, BRCA1, BRCA2, Anti-Müllerian hormone, Ovarian response, Ovarian reserve

## Abstract

This study aimed to investigate whether female BRCA1- and BRCA2 mutation carriers have a reduced ovarian reserve status, based on serum anti-Mullerian hormone (AMH) levels, antral follicle count (AFC) and ovarian response to ovarian hyperstimulation. A prospective, multinational cohort study was performed between October 2014 and December 2019. Normo-ovulatory women, aged 18–41 years old, applying for their first PGT-cycle for reason of a BRCA mutation (cases) or other genetic diseases unrelated to ovarian reserve (controls), were asked to participate. All participants underwent a ICSI-PGT cycle with a long-agonist protocol for controlled ovarian hyperstimulation. Linear and logistic regression models were used to compare AMH, AFC and ovarian response in cases and controls. Sensitivity analyses were conducted on BRCA1- and BRCA2 mutation carrier subgroups. Thirty-six BRCA mutation carriers (18 BRCA1- and 18 BRCA2 mutation carriers) and 126 controls, with mean female age 30.4 years, were included in the primary analysis. Unadjusted median AMH serum levels (IQR) were 2.40 (1.80–3.00) ng/ml in BRCA mutation carriers and 2.15 (1.30–3.40) ng/ml in controls (*p* = 0.45), median AFC (IQR) was 15.0 (10.8–20.3) and 14.5 (9.0–20.0), *p* = 0.54, respectively. Low response rate was 22.6% among BRCA mutation carriers and 9.3% among controls, *p* = 0.06. Median number of retrieved oocytes was 9 (6–14) in carriers and 10 (7–13) in controls, *p* = 0.36. No substantial differences were observed between BRCA1- and BRCA2 mutation carriers. Based on several biomarkers, no meaningful differences in ovarian reserve status were observed in female BRCA mutation carriers compared to controls in the context of ICSI-PGT treatment.

**Supplementary Information:**

The online version contains supplementary material available at 10.1007/s43032-022-00997-w.

## Introduction

BRCA genes have a function in the ataxia-telangiectasia mutated (ATM) mediated DNA double-strand breaks (DSB) repair pathway [1–3]. Loss of function can cause inadequate repair and accumulation of DNA damage that predisposes to carcinogenesis and apoptosis in rapidly dividing cells. This condition makes BRCA mutation carriers highly susceptible to early-onset breast and ovarian cancer [4, 5]. DNA damage in resting cells, such as the oocyte of the primordial follicle, accumulates over time and is believed to cause the effects of ageing on oocyte intregity [6]. This natural process contributes to the gradual decline in reproductive capability and the occurrence of natural infertility approximately 10 years before menopause occurs [7, 8]. A reduced DNA integrity in BRCA mutated cells could hypothetically lead to accelerated follicular loss, resulting in a reduced ovarian reserve and premature ovarian insufficiency in BRCA mutation carriers [9, 10]. In vitro studies have demonstrated high expression of BRCA genes (primarily BRCA1) in germ cells and blastocysts, suggesting a potential role in gametogenesis and embryogenesis [11], which could reasonably have a negative effect on fecundity in BRCA mutation carriers as well.

Anti-Müllerian hormone (AMH) is considered to be a quantitative biomarker for reproductive lifespan and ovarian reserve [12]. AMH is produced by the small antral follicles and preantral follicles, and serum levels are found to be proportional to the number of primordial follicles in the ovaries [13]. Changes in AMH levels with age are seen before any other signs of the ovarian ageing process become notable, such as cycle length changes and infertility, which implies that AMH could specify a woman’s reproductive age more realistically than chronological age alone [14]. The antral follicle count (AFC) also indirectly reflects the number of remaining primordial follicles and is another quantitative test for describing ovarian reserve status [13]. Both AMH and AFC have demonstrated to have strong correlations with the number of dominant follicles in response to ovarian stimulation for IVF/ICSI cycles [15]. Therefore, ovarian response to ovarian hyperstimulation can be used as a proxy variable of ovarian reserve status as well.

Current evidence regarding the potential existence of a reduced ovarian reserve in BRCA mutation carriers contains conflicting results. Several studies demonstrated significantly lower AMH serum levels in BRCA1 mutation carriers [10, 16–19] or BRCA2 mutation carriers [20] compared to controls. Other studies did not show any (statistically significant) differences in AMH serum levels [21–25]. An association between BRCA mutations and low ovarian response rate was found in women with breast cancer undergoing ovarian stimulation for the purpose of fertility preservation ([9, 26]. However, these studies included only symptomatic carriers and other studies reported conflicting results [23, 24]. A significant difference in mean age of natural menopause between BRCA1- and BRCA2 mutation carriers and non-carriers was also reported in three studies [27–29], but other large studies could not confirm these findings [30, 31].

If BRCA mutation carriers would indeed be susceptible to earlier menopause and low response to ovarian stimulation, improvements in preventative reproductive health care and family planning for BRCA mutation positive women could be envisaged. Therefore, the aim of this study is to assess ovarian ageing status in BRCA mutation carriers by measuring serum AMH levels, AFC and ovarian response to ovarian hyperstimulation.

## Materials and Methods

### Study Population and Participants

This multicenter, multinational, observational prospective cohort study included normo-ovulatory BRCA mutation carriers (BRCA1 gene-mutation #604,370 or BRCA2 gene-mutation #612,555) and controls between 18 and 41 years old, during their first intracytoplasmic sperm injection (ICSI) and preimplantation genetic testing (PGT) cycle. Controls applied for PGT for other genetic indications that were considered to be unrelated to ovarian reserve and were not suspected for a BRCA mutation carriership (see supplementary A for a full list of diagnoses). Patients were recruited between October 2014 and December 2019 from the list of PGT indicated couples in four participating academic hospitals in the Netherlands (University Medical Centre Utrecht, Maastricht University Medical Centre, University Medical Centre Groningen and Amsterdam University Medical Centre (location AMC)) and one participating academic hospital in Belgium (Universitair Ziekenhuis Brussel). Subjects that had a history of ovarian surgery, chemo-therapy or radiation therapy to the pelvis/lower abdomen/total body radiation were considered ineligible. Other exclusion criteria were known female endocrine or autoimmune abnormalities, polycystic ovarian syndrome (PCOS; [32]), known HIV infection, known genetic abnormalities suspected for subfertility (e.g. Turner syndrome, Fragile X syndrome) and/or PGT requested for structural chromosomal abnormalities.

### Measurements

Baseline characteristics and data concerning PGT/ICSI cycles were collected by local research nurses for all patients meeting the inclusion criteria. Both exposed and unexposed participants were treated according to local protocols for controlled ovarian stimulation and oocyte retrieval. All subjects received a long agonist protocol for luteinizing hormone (LH) suppression. The majority of cycles were combined with oral contraceptive pretreatment. Exogenous ovarian stimulation, applying a daily dosage of 150–225 IU recombinant follicle stimulating hormone (rFSH) or highly purified human menopausal gonadotrophin (hMG), was started when downregulation was achieved. During the uterine bleeding, prior to the start of ovarian stimulation, subjects provided a blood sample for storage at − 80 °C and an AFC was assessed by experienced and properly trained physicians, applying a method described before [33]. All ovarian follicles measuring 2–10 mm in mean diameter in each ovary were considered using a standard transvaginal sonography (TVS)-based measurement. At the end of the study, stored serum aliquots were thawed and serum AMH levels were determined simultaneously in a single expert laboratory using an ultra-sensitive enzyme linked immunosorbent assay (ELISA) on a robotic platform (Elecsys AMH Plus, Cobas, [34]). The minimum detectable concentration was 0.03 ng/mL with an intermediate coefficient of variation ≤ 8%.

After reaching known criteria [35], final oocyte maturation triggering was applied using urinary or recombinant hCG, and 34–38 h later, oocyte retrieval was performed. Low ovarian response (‘low response’) was defined as less than 4 oocytes at retrieval or cancellation due to insufficient follicle growths (i.e. < 4 dominant follicles sized > 14 mm growing). Expected and unexpected low response were determined based on AMH serum levels and AFC, applying threshold values as earlier described in the OPTIMIST-trial (i.e. low response was expected in females with AMH < 0.96 ng/ml or AFC 0–7) [36]. Follicular punction was cancelled due to hyper response in subjects with either > 20 follicles sized > 10 mm with estradiol levels exceeding 15,000 pmol/L or more than 35 follicles > 10 mm. Patients with > 15 retrieved oocytes at follicle aspiration were classified as hyper responders as well.

The number of mature (MII) oocytes was determined during the ICSI procedure. Fertilization and embryo biopsies were executed following local standard regimens.The number of embryos that was biopsied was divided by the number of retrieved oocytes and presented as a fraction, labelled as ‘fraction biopsied’. The outcome of the first (fresh or cryo) transfer with an unaffected embryo regarding the genetic target disease was included. All performed embryo transfers were (elective) single embryo transfers (SET).

### Statistical Analysis

Clinical characteristics, serum AMH, AFC and ovarian response were compared based on the presence of BRCA mutation. Data are presented as mean (SD; standard deviation), median (IQR; interquartile range) or number (percentage) based on distribution. Differences were analysed using chi-square test or Fisher’s exact tests for categorical variables and independent two-sample *t*-test or Mann–Whitney *U*-test for continuous variables.

Linear regression analyses were performed to compare log-transformed serum AMH and square-root transformed AFC in BRCA mutation carriers and controls. Adjustments were made for age, body-mass index, smoking (yes/no), parity (nulliparous/multiparous) and oral contraceptive use in downregulation (yes/no). Beta coefficients for AMH values were retransformed into the original scale and presented as geometric mean ratio (GMR). MANCOVA was used to globally analyse overall response ovarian hyperstimulation in continuous outcomes (i.e. number of retrieved oocytes, mature oocytes and fraction biopsied). Low response rate and pregnancy rate were evaluated in both subgroups using logistic regression models. Variables were square-root transformed in case of non-normal distribution. Models were controlled for the above-mentioned adjustments extended with cumulative dose FSH and type gonadotropin administered (rFSH/uFSH). For both primary (AMH) and secondary (AFC, ovarian response) outcome measurements, sensitivity analyses comparing BRCA1- and BRCA2 subgroups to controls and analyses excluding female non-BRCA carriers in the control-group were conducted. Results were presented as beta coefficients in continious variables and exp(B)/odds-ratio’s for categorical variables, along with their corresponding 95% confidence interval and level of significance.

Subjects with missing blood samples were excluded from the study and replaced by a new subject if possible in order to gain sufficient power on the primary outcome. Women who received < 150 IU/day gonadotropin for ovarian stimulation were excluded in the primary analysis on response ovarian hyperstimulation to strive for a homogenous study population with optimal ovarian stimulation. These excluded subjects, as well as other participants with protocol violations (i.e. BMI > 35 kg/m^2^, wrong timing in AFC assessment or blood sample collection and PGT requested for reciprocal translocation), were additionally analysed in secondary (full) analyses.

Multiple imputation by chained equations was used to correct for a single missing BMI value. All analyses were executed using IBM SPSS statistics 25.0.0.2. (32 bits). *P*-values < 0.05 were considered statistically significant.

Power calculation was based on the hypothesis that women with a deleterious BRCA mutation have lower levels of AMH compared to normal controls. A difference in serum AMH level of 0.90 ng/ml (SD ± 1.6 ng/ml) was considered to be clinically relevant as this would suggest a potential effect size of approximately 5 years in the timing of menopause. With *α* < 0.05, *β* = 0.80 and relevant effect size (Cohen’s d) of 0.56 (= 0.9/1.6), including 34 BRCA mutation carriers and 91 controls would be sufficient to detect a difference of the aforementioned magnitude. Within the sample size calculation, potential confounders (such as female age) were not considered, as this would have led to the use of an age-restricted or age-matched design, which automatically will affect the power of the study in an unfavourable way.

## Results

**Fig. 1:**
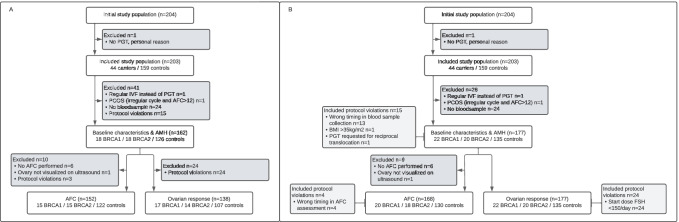
Study flowchart. **a** Flowchart for primary analyses, including only participants who completely fulfilled all in/exclusion criteria. **b** Flowchart for secondary (full) analyses, including subjects with protocol violations PGT, pre-implantation genetic testing; PCOS, polycystic ovarian syndrome; AMH, anti-Mullerian hormone; AFC, antral follicle count; FSH, follicle-stimulating hormone

A total of 204 women was willing to participate and included in the study. One woman did not start with PGT cycle due to personal reasons and was subsequently excluded from the initial study population. Accordingly, 203 participants (44 BRCA mutation carriers, 159 controls) were included in the BROCA2-study. The presented analyses of primary and secondary outcomes were conducted in subjects who completely fulfilled all inclusion criteria (see Fig. 1a: flowchart).

### Baseline Characteristics

As presented in Table 1, 36 BRCA mutation carriers (18 BRCA1 and 18 BRCA2) and 126 controls were included in baseline analysis. Among the included controls, 73 subjects (57.9%) were affected with genetic abnormalities other than BRCA-mutations (female non-BRCA carriers), unsuspected for potential effects on fertility or ovarian ageing). The most prevalent genetic diagnoses in female non BRCA carriers were Huntington disease (*n* = 10), haemophilia (*n* = 5), cystic fibrosis (*n* = 4) and neurofibromatosis (*n* = 4) (full list of diagnoses is enclosed as Supplementary A). The other 53 controls (42.1%) requested PGT because of a male partner with a genetic disorder (non-BRCA carriers). Mean age was 30.4 years in both carriers and controls. All included participants had regular menstrual cycles, with mean cycle lengths of 28 days. A higher percentage of BRCA mutation carriers was nulligravid and nulliparous (83.3% carriers vs 68.3% controls and 80.6% carriers vs 64.3% controls, respectively); however, this was only statistically different in nulligravid BRCA1 mutation carriers compared to controls.

**Table 1:** Baseline characteristics

Baseline characteristics	BRCA mutation carrier (*n* = 36)	Sig.*	BRCA1 mutation carrier (*n* = 18)	Sig.*	BRCA2 mutation carrier (*n* = 18)	Sig.*	Control (*n* = 126)
Female age (years)	30.4 (± 2.8)	0.80	31.0 (± 2.9)	0.32	29.7 (± 2.6)	0.55	30.4 (± 4.0)
Female BMI (kg/m^2^)	22.7 (± 3.4)	0.27	23.1 (± 4.3)	0.49	22.4 (± 2.2)	0.34	23.6 (± 3.9)
Caucasian	36 (100%)	0.20	18 (100%)	0.60	18 (100%)	0.60	118 (93.7%)
Smoking	2 (5.6%)	1.000	0 (0%)	0.60	2 (11.1%)	0.63	9 (7.1%)
Alcohol	18 (50%)	0.93	9 (50%)	0.95	9 (50%)	0.95	62 (49.2%)
Drugs	1 (2.8%)	0.40	0 (0%)	1.000	1 (5.6%)	0.23	1 (0.8%)
**PGT indication**							
Female BRCA1 mutation carrier	18 (50%)		18 (100%)		NA		N/A
Female BRCA2 mutation carrier	18 (50%)		NA		18 (100%)		N/A
Female non BRCA mutation carrier	N/A		NA		NA		73 (57.9%)
Male non BRCA mutation carrier	N/A		NA		NA		53 (42.1%)
**Cycle information**							
Age at menarche (years)	13.3 (± 1.4)	0.23	13.5 (± 1.4)	0.10	13.0 (± 1.4)	0.87	13.0 (± 1.4)
Mean length of menstrual cycle (days)	28.0 (± 1.3)	0.25	28.2 (± 1.1)	0.61	27.7 (± 1.3)	0.25	28.4 (± 1.7)
Reproductive history							
Subfertility	3 (8.3%)	0.71	2 (11.1%)	0.36	1 (5.6%)	1.000	8 (6.3%)
Prior fertility treatment	2 (5.6%)	0.21	2 (11.1%)	0.08	0 (0%)	1.000	2 (1.6%)
Nulliparity	30 (83.3%)	0.08	16 (88.9%)	0.07	14 (77.8%)	0.41	86 (68.3%)
Nulligravidity	29 (80.6%)	0.07	16 (88.9%)	**0.03**	13 (72.2%)	0.51	81 (64.3%)
Live birth	5 (13.9%)	0.11	2 (11.1%)	0.24	3 (16.7%)	0.56	34 (27%)
**Family history**							
Early menopause (age < 40 years)	1 (2.8%)	0.22	0 (0%)	NA	1 (5.6%)	0.13	0 (0%)
Subfertility^a^	3 (8.3%)	0.38	1 (5.6%)	0.56	2 (11.1%)	0.21	5 (4.0%)
Menopausal age mother (years)	49.1 (± 5.8)	0.47	51.5 (± 6.3)	0.53	47.9 (± 5.2)	0.17	50.4 (± 4.4)
Breast or ovarian cancer	33 (91.7%)	** < 0.001**	16 (88.9%)	** < 0.001**	17 (94.4%)	** < 0.001**	7 (5.7%)

Values presented as number (%) in categorical variables, values presented as mean (SD) in continuous variables. *BMI*, body-mass index; *PGT*, pre-implantation genetic testing; *EUG*, extra uterine gravidity; *no*, number; *NA*, not applicable; *SD*, standard deviation; *CI*, confidence interval. *P-values comparing values in carriers vs controls, calculated using Fisher’s exact/chi-square or Mann–Whitney *U.*^a^Subfertility issues in mother, sister and/or aunt (mothers side) necessitating referral for fertility investigation or treatment

### AMH Serum Levels and AFC

**Fig. 2:**
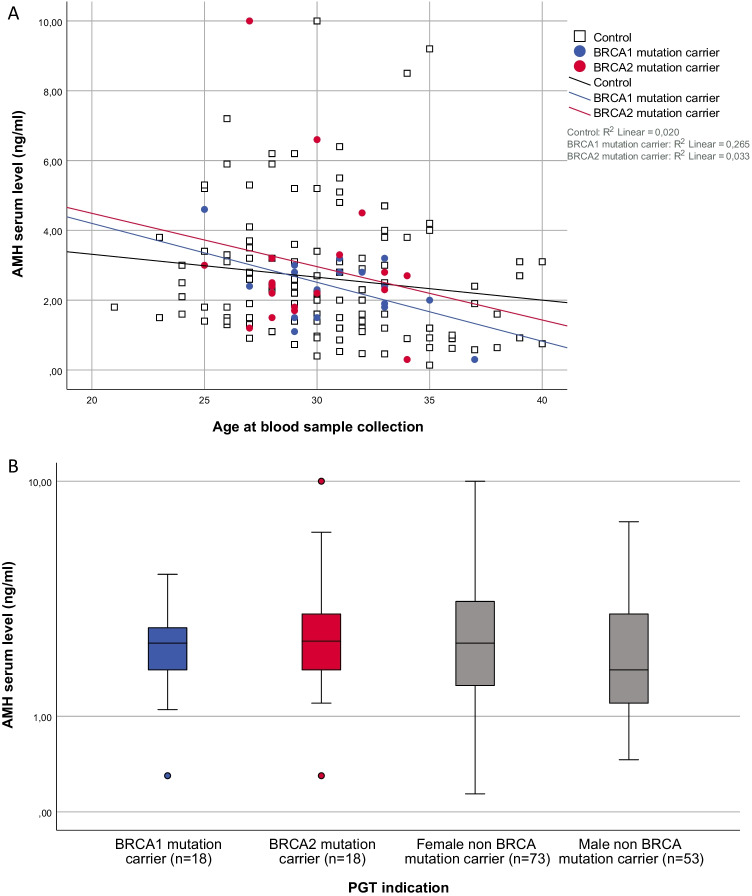
Scatterplot and boxplot representing serum AMH levels. PGT, preimplantation genetic testing; AMH, anti-Mullerian hormone. **a** Scatterplot representing serum AMH level (ng/ml) on a logarithmic scale by age (in years) in BRCA1 mutation carriers (*n* = 18, blue dots), BRCA2 mutation carriers (*n* = 18, pink dots) and controls (*n* = 126, white squares). The visualized lines are fit lines for BRCA1 mutation carriers (blue regression line, AMHBRCA1 = 7.58–0.17*age), BRCA2 mutation carriers (pink regression line, AMHBRCA2 = 7.54–0.15*age) and controls (black/dotted regression line, AMHcontrol = 4.63–0.07*age). **b** Boxplot representing median and interquartile ranges of female AMH serum levels (ng/ml) on a logarithmic scale in couples with PGT indication due to BRCA1-, BRCA2- and female/male non-BRCA gene mutations. BRCA1 mutation carriers are presented in blue, BRCA2 mutation carriers in pink and controls in grey

Unadjusted median AMH serum levels (IQR) were 2.40 (1.80–3.00) ng/ml in BRCA mutation carriers and 2.15 (1.30–3.40) ng/ml in controls, *p* = 0.45. No significant effect of carriers status on AMH serum levels was identified using the linear regression model (unadjusted GMR 1.03, 95% *CI* 0.34–3.19, *p* = 0.56, fully adjusted GMR 1.01, 95% *CI* 0.33–3.10, *p* = 0.94). The boxplots and scatterplot depicted comparative distribution among all subgroups (see Table 2 and Fig. 2).

**Table 2:** Serum AMH levels and antral follicle count (AFC) in BRCA mutation carriers and controls

	BRCA mutation carrier (*n* = 36)	Sig.*	BRCA1 mutation carrier (*n* = 18)	Sig.*	BRCA2 mutation carrier (*n* = 18)	Sig.*	Control (*n* = 126)
Unadjusted serum level AMH (ng/ml) median (IQR)	2.40 (1.80–3.00)	0.45	2.40 (1.73–2.85)	0.85	2.45 (1.78–3.23)	0.34	2.15 (1.35–3.40)
**Linear regression model**							
Geometric mean ratio (95% *CI*)							
BRCA carrier status, unadjusted	1.03 (0.34–3.19)	0.56	1.00 (0.31–5.37)	0.99	1.07 (0.34–3.49)	0.39	
BRCA carrier status, age-adjusted	1.03 (0.34–3.19)	0.55	1.02 (0.32–3.25)	0.83	1.05 (0.33–3.39)	0.49	
BRCA carrier status, fully-adjusted^b^	1.01 (0.33–3.10)	0.94	0.98 (0.30–3.82)	0.76	1.03 (0.32–3.32)	0.67	
	BRCA mutation carrier (*n* = 30)	Sig.*	BRCA1 mutation carrier (*n* = 15)	Sig.*	BRCA2 mutation carrier (*n* = 15)	Sig.*	Control (*n* = 122)
Unadjusted AFC median (IQR)	15.0 (10.8–20.3)	0.54	14.0 (10.0–20.0)	0.86	15.0 (12.0–28.0)	0.27	14.5 (9.0–20.0)
**Linear regression model**							
Coefficient^a^, β (95% *CI*)							
BRCA carrier status, unadjusted	0.18 (− 0.26–0.61)	0.42	− 0.09 (− 0.67–0.50)	0.77	0.44 (− 0.14–1.03)	0.14	
BRCA carrier status, age-adjusted	0.17 (− 0.26–0.60)	0.43	− 0.08 (− 0.66–0.49)	0.77	0.43 (− 0.15–1.01)	0.14	
BRCA carrier status, fully-adjusted^b^	0.17 (− 0.28–0.62)	0.46	− 0.09 (− 0.69–0.52)	0.78	0.42 (− 0.18–1.01)	0.17	

*AMH*, anti-Mullerian hormone; *AFC*, antral follicle count; *IQR*, interquartile range; *CI*, confidence interval. Median AFC (2–10 mm) in both ovaries assessed using a standard transvaginal sonography (TVS). *P-values comparing AMH or AFC in carriers vs controls, calculated using Mann Whitney *U.* ^a^Estimated coefficient (β) of BRCA carrier status on the natural logarithm of AMH serum levels, retransformed into the original scale (Exp(β)) or estimated coefficient of BRCA carrier status on square-root transformed AFC. ^b^Adjusted for age, BMI, gravidity, smoking and oral contraceptive use in downregulation

After excluding 9 women who had no (or incorrectly timed) AFC assessment, AFC was analysed in 30 carriers (15 BRCA1- and 15 BRCA2 mutation mutation carriers) and 122 controls (Table 2). Unadjusted median AFCs (IQR) were 15.0 (10.8–20.3) in carriers and 14.5 (9.0–20.0) in controls (*p* = 0.54). The estimated coefficient of BRCA carrier status on square-root transformed AFC was 0.12 (95% *CI* − 0.18–0.43, *p* = 0.42) for unadjusted analysis and 0.10 (95% *CI* − 0.21–0.41, *p* = 0.54) in fully-adjusted analysis.

No differences were observed between BRCA1- and BRCA2-mutation carriers in comparison to controls in the performed sub analyses.

### Ovarian Response and Pregnancy

Table 3 presents data regarding ICSI/PGT cycle in the 138 participants (31 BRCA mutation carriers, 107 controls) who received 150–225 IU FSH as a daily dosage. In the majority of protocols, rFSH was used as stimulating medicine. The mean number of days stimulated was ~ 11 in both groups and carriers received a mean cumulative dose FSH of 1925 ± 543 IU and controls 1959 ± 567 IU, *p* = 0.78.

**Table 3:** ICSI/PGT cycle and ovarian response in BRCA mutation carriers and controls

ICSI/PGT cycle	BRCA mutation carrier (*n* = 31)	Sig.*	BRCA1 mutation carrier (*n* = 17)	Sig.*	BRCA2 mutation carrier (*n* = 14)	Sig.*	Control (*n* = 107)
**Received treatment**							
Long-agonist with oral contraceptive	24 (77.4%)	**0.03**	14 (82.4%)	0.21	10 (71.4%)	**0.04**	98 (91.6%)
Stimulating medicine		**0.01**		0.27		**0.01**	
rFSH	21 (67.7%)		13 (76.5%)		8 (57.1%)		93 (86.9%)
uFSH	10 (32.3%)		4 (23.5%)		6 (42.9%)		14 (13.1%)
Start dose FSH							
150/day	22 (71.0%)	0.56	12 (70.6%)	0.68	10 (71.4%)	0.77	70 (65.4%)
187.5/day	1 (3.2%)	0.40	0 (0%)	1.000	1 (7.1%)	0.22	1 (0.9%)
200/day	0 (0%)	1.000	0 (0%)	1.000	0 (0%)	1.000	1 (0.9%)
225/day	8 (25.8%)	0.47	5 (29.4%)	0.79	3 (21.4%)	0.55	35 (32.7%)
Dose adjustments (FSH)							
Dose increased	6 (19.4%)	**0.02**	4 (23.5%)	**0.02**	2 (14.3%)	0.19	5 (4.7%)
Dose decreased	0 (0%)	NA	0 (0%)	NA	0 (0%)	NA	0 (0%)
Received cumulative dose FSH	1925 (± 543)	0.78	2122 (± 778)	0.59	1769 (± 270)	0.43	1959 (± 567)
Number of days stimulated	11.3 (± 2.0)	0.78	11.5 (± 2.8)	0.70	11.1 (± 1.9)	0.82	11.1 (± 2.0)
Hyper response	5 (16.1%)	0.66	1 (5.9%)	0.30	4 (28.6%)	0.48	21 (19.6%)
- Cancelled follicular punction	1 (3.2%)		0 (0%)		1 (7.1%)		4 (3.7%)
- > 15 oocytes at follicle aspiration	4 (12.9%)		1 (5.9%)		3 (21.4%)		17 (15.9%)
**Low response**							
Low response	7 (22.6%)	0.06	4 (23.5%)	0.10	3 (21.4%)	0.17	10 (9.3%)
Unexpected low response^a^	5 (71.4%)		3 (75.0%)		2 (66.7%)		3 (30.0%)
- Cancelled oocyte retrieval	5 (16.1%)		3 (75.0%)		2 (66.7%)		6 (5.6%)
- < 4 oocytes at retrieval	2 (6.5%)		1 (25.0%)		1 (33.3%)		4 (3.7%)
Odds-ratio (95% *CI*) for low response							
Unadjusted	2.83 (0.98–8.20)	0.06	2.99 (0.82–10.91)	0.10	2.65 (0.63–11.09)	0.18	
Age-adjusted	3.16 (1.04–9.67)	**0.04**	2.91 (0.77–11.00)	0.12	3.56 (0.79–16.07)	0.10	
Fully-adjusted^b^	3.47 (0.85–14.21)	0.08	2.91 (0.50–16.93)	0.23	3.98 (0.66–24.18)	0.13	
**Oocyte retrieval performed**	**BRCA mutation carrier (*n* = 25)**	Sig.*	**BRCA1 mutation carrier (*n* = 14)**	Sig.*	**BRCA2 mutation carrier (*n* = 11)**	Sig.*	**Control (*n* = 96)**
	80.6%		82.4%		78.6%		89.7%
Total oocytes	9 (6–14)	0.36	9 (6–12)	0.32	9 (4–16)	0.75	10 (7–13)
Mature (MII) oocytes	7 (5–10)	0.23	7 (5–10)	0.33	7 (4–10)	0.41	8 (6–12)
Fraction biopsied^c^	0.59 (± 0.24)	0.51	0.56 (± 0.20)	0.29	0.63 (± 0.29)	0.92	0.63 (± 0.23)
Mancova, Wilk’s Lambda sig.^d^							
Unadjusted		0.72		0.55		0.90	
Age-adjusted		0.74		0.63		0.87	
Fully-adjusted^b^		0.84		0.76		0.68	
**Pregnancy per started cycle**	**BRCA mutation carrier (*n* = 31)**	Sig.*	**BRCA1 mutation carrier (*n* = 17)**	Sig.*	**BRCA2 mutation carrier (*n* = 14)**	Sig.*	**Control (*n* = 107)**
Cycles with embryo transfer (SET)	22 (71.0%)	0.75	12 (70.6%)	0.77	10 (71.4%)	1.000	79 (73.8%)
Pregnancy	8 (25.6%)	0.78	4 (23.5%)	1.000	2 (14.3%)	1.000	25 (23.4%)
Biochemical pregnancy, miscarriage or molar pregnancy	4 (12.9%)		2 (11.8%)		0 (0%)		7 (6.5%)
Ongoing clinical pregnancy^e^	4 (12.9%)		2 (11.8%)		2 (14.3%)		18 (16.8%)
Odds-ratio (95% *CI*) for ongoing pregnancy							
Unadjusted	0.81 (0.28–2.32)	0.69	0.71 (0.15–3.37)	0.66	0.88 (0.18–4.30)	0.88	
Age-adjusted	0.81 (0.28–2.33)	0.70	0.66 (0.14–3.18)	0.61	0.96 (0.20–4.76)	0.96	
Fully-adjusted^b^	1.05 (0.30–3.64)	0.94	1.02 (0.17–6.18)	0.98	1.43 (0.23–8.83)	0.70	

*rFSH/uFSH*, recombinant/urinary follicle-stimulating hormone; *CI*, confidence interval; *SET*, single embryo transfer; *NA*, not applicable. Values presented as number (%) in categorical variables, values presented as mean (SD) or median (IQR) in continuous variables. *P-values comparing values in carriers vs controls, calculated using Fisher’s exact/chi-square or Mann–Whitney *U*/*T*-test. ^a^Low response was expected in females with *AMH* < 0.96 ng/ml or AFC 0–7. ^b^Adjusted for age, BMI, gravidity, smoking, oral contraceptive use in downregulation, type- and cumulative dosage of administered gonadotropin. ^c^Number of embryos that was biopsied, divided by the number of retrieved oocytes. ^d^Level of significance for variance in total oocytes, mature oocytes or fraction biopsied, explained by BRCA carrier status. ^e^Ongoing clinical pregnancy with foetal heartbeat at 7 weeks of gestation

There were 17 low responders in this study population (8/17 unexpected), among those were 7 BRCA mutation carriers (22.6%) and 10 controls (9.3%), *p* = 0.06. Odds-ratio for low response in BRCA mutation carriers was unadjusted 2.83 (95% *CI* 0.98–8.20, *p* = 0.055), age-adjusted 3.16 (95% *CI* 1.04–9.67, *p* = 0.04) and fully-adjusted 3.47 (95% *CI* 0.87–14.21, *p* = 0.08).

Within the 121 participants (25 BRCA mutation carriers, 96 controls) who had a follicular aspiration, the median number of retrieved oocytes was 9 (6–14) in carriers and 10 (7–13) in controls, *p* = 0.36. The number of mature (MII) oocytes was 7 (5–10) in carriers and 8 (6–12) in controls, *p* = 0.23. In the adjusted model, no statistical significant difference in retrieved (mature) oocytes or fraction embryo’s biopsied was found (fully adjusted *p* = 0.84, mancova).

The pregnancy rate per started cycle was 25.6% in 31 carriers and 23.4% in 107 controls, *p* = 0.78. Unadjusted odds-ratio for ongoing clinical pregnancy (i.e. pregnancy with foetal heartbeat at 7 weeks of gestations) in carriers was 0.81 (95% *CI* 0.28–2.32, *p* = 0.69), fully-adjusted 1.05 (95% *CI* 0.30–3.64, *p* = 0.94).

The performed sensitivity analyses, excluding female non-BRCA mutation carriers in the control group (i.e. including only *n* = 53 healthy carriers with PGT indication due to male genetic factors) did not produce statistically significant differences, data are prensented in Supplementary B.

### Full Analyses

In secondary (full) analyses, 13 participants with protocol-deviated timing in blood sample collection (i.e. prior to PGT cycle or after start stimulating treatment), 4 participants with protocol-deviated timing in AFC assessment (i.e. before start treatment), 24 participants who received < 150 IU FSH/day during ovarian hyperstimulation and 2 subjects with other protocol violations (i.e. BMI > 35 kg/m^2^ and PGT requested for reciprocal translocation) were additionally analysed (see Flowchart 1b and Supplementary C). Compared to initial analyses, no differences in primary and secondary outcomes were observed.

## Discussion

### Main Finding

This study evaluated the effect of a BRCA mutation on quantitative ovarian reserve status and revealed that AMH levels in BRCA mutation carriers do not clearly differ from those found in controls undergoing the same type of assisted reproduction treatment. In addition, AFC and ovarian response were included as other markers for ovarian reserve and no statistically significant differences were observed.

### Present Findings in View of Existing Literature

#### AMH

Unadjusted AMH values and regression equations were very similar in the evaluated subgroups and variation in AMH serum levels could not be attributed to BRCA carrier status. Main findings from other studies that evaluate BRCA mutations and AMH serum levels range from significantly lower (adjusted) AMH serum levels up to no significant differences [10, 17–25, 37–39]. Studies are often heterogeneous in methodological aspects and, in the absence of appropriate adjustments, variance in studied participants (e.g. age at sampling, chronic ovarian dysfunction such as PCOS, use of hormonal contraceptives and breast cancer affected or not) could interfere with the observed results. In a recent meta-analysis [40], nine papers reporting on AMH serum levels [10, 17, 18, 20–23, 23, 25] were included and no overall significant association between BRCA mutation status and AMH was found.

#### AFC

Three recent studies incorporated AFC measurements in their comparison of ovarian reserve in BRCA carriers and controls [19, 24, 25]. Even though Grynberg et al. were the only one to describe the method for AFC assessment, all reported counts were quite comparable and no study reported a statistically significant difference in AFC. This is equivalent to our results and supports the overall impression of an unaffected quantitative ovarian reserve status in BRCA mutation carriers.

#### Low Response

In the evaluation of response to ovarian hyperstimulation, a significantly higher age-adjusted odds-ratio for low response in the carriers was observed. Starting doses were similar in BRCA mutation carriers and controls, but BRCA mutation carriers required more often a dose increasement, which may suggest a difference in sensitivity to FSH of the antral follicle. However, the high percentage of unexpected low responding BRCA mutation carriers (5/7, 71.45%) could also indicate that BRCA mutation carriers were not all maximally stimulated. The starting dose for all participants was determined by the local physician, based on experience, ovarian reserve testing and with the principle that using a dosage range of 150–225 IU will maximize ovarian response in the vast majority of cases. Indeed, in PGT treatment, a maximal number of oocytes is aimed for. Still, in cases with a high ovarian reserve maximal stimulation may have been refrained from in view of safety risks. All of our cases with an ovarian reserve test indicating a low response certainly will certainly have been selected for a dosage of 225 IU daily. Applying dosages of over 225 IU in predicted low responders has never been scientifically substantiated [41]. Finally, one may keep in mind that studies using a fixed dose of 150 IU FSH have revealed that serum FSH levels relate poorly to ovarian response category, while in contrast, the effect of AMH level was much larger [42]. From the ideal study perspective, it would have been more desirable to have applied a 300 IU dosage for everybody in order to be sure of maximal stimulation, making the comparison as clean as possible. With the applied FSH dosing based on local protocols but with the purpose of maximizing response if sufficiently safe, we still believe to have created the best possible comparison within ethical limits.

Two small studies (*n* = 12 and *n* = 10 carriers) reported high prevalences of low responders in the BRCA mutation carrier subgroup compared to the control group (33% in BRCA mutation carriers versus 3%, *p* = 0.01 and 40% versus 11% *p* = 0.15, respectively) [9, 23]. The two other studies that reported on low response rate in female BRCA mutation carriers were better powered (*n* = 43 and *n* = 63 carriers) and reported highly comparable low response rates in carriers and controls (7–8% in carriers and 6–9% in controls) [43, 44]. With 31 included subjects with BRCA-mutations in the current BROCA2 study, a trend towards low ovarian response may have been the result of insufficient sample size.

#### Oocyte Yield

The mean number of yielded (mature) oocytes and fraction embryo’s biopsied were highly comparable for BRCA mutation carriers and controls and no statistically significant difference was found in the present study. Similarly to studies on AMH serum levels, reported results on ovarian response are inconsistent [9, 19, 23–26, 43, 44]. Sample size, eligibility criteria and stimulation protocols all vary and only two studies adjusted for dosage of gonadotropin administered [43, 44]. The previously mentioned meta-analysis included four papers and did not find any statistically significant difference in ovarian response [40].

#### BRCA1- and BRCA2 Mutation Carriers

The results of the conducted sensitivity analyses in this cohort did not demonstrate substantial differences in quantitative ovarian reserve status between the included BRCA1- and BRCA2 mutation carriers, although not enough carriers were enrolled to perform a powered subgroup analysis for this specific outcome. In some preceding studies, differences in ovarian reserve were only detected in BRCA1 mutation carriers [9, 10, 16, 18, 19, 26, 44], while other studies reported differences in ovarian reserve that only existed in BRCA2 mutation carriers [20, 38]. There is a biologic rationale, supported by discoveries in mice, for a distinct molecular function of BRCA1- and BRCA2 genes in DNA repair pathway [6, 10, 45, 46], However, the hypothesized more critical role for BRCA1 genes in preventing oocyte depletion [47] could not be confirmed by previous datapooling [40] and similar to the ambigious results on the impact of BRCA genes on quantitative ovarian reserve, there is no consensus on potential existing differences in ovarian response within BRCA-mutation carriers.

#### Pregnancy

Ongoing pregnancy rates were comparable among carriers and controls. This study was not powered to detect a difference in pregnancy outcomes, yet this is in line with other studies on the impact of AMH on reproductive outcome in BRCA carriers where reproductive outcomes in terms of miscarriage and live birth rates were similar [38, 48, 49]. Other studies compared parity and the prevalence of fertility problems between BRCA mutation carriers and an unaffected population and mostly did not report statistically significant differences [11, 16, 18, 22, 28–31, 50–52]. So all together, no obvious evidence of a decreased reproductive potential in BRCA carriers exists.

### Strengths and Limitations

The main strength of this study was the use of well-validated markers for ovarian reserve that were addressed. Furthermore, we had a wide range of case characteristics available, which allowed a reliable extensive evaluation of potential confounders as documented in literature. In the analyses, age was a statistically significant covariate in all models and smoking was statistically significant in the regression model for AMH. Nevertheless, no substantiated evidence for confounders in this study were found, as no significant differences in baseline characteristics were observed (except for the expected discrepancy in family history of breast cancer). Considering the population risk of 0.2–1% to carry a BRCA-mutation in the western population [53], the chance of including a control participant with a BRCA mutation was low. Finally, all AMH samples were analysed simultaneously in a single expert laboratory.

The main limitation of this study involves the sample size. The aimed inclusion of 34 BRCA mutation carriers was obtained, and according to the sample size calculation that was applied, the number of included participants in this study was large enough to detect a clinically relevant diffence in AMH. However, in previous studies, the observed associations between mutation carrier status and reduced ovarian reserve have been rather small. This obviously would require larger sample sizes to detect such small differences. Another limitation would be the relatively large number of participants (*n* = 24) that were to be excluded due to no or wrongfully taken blood samples. Nevertheless, analyses conducted on those participants did not demonstrate any specific patient characteristic that could have been a manifestation of selection bias. Besides, in full analyses, all participants with protocol violations were additionally analysed and no (statistically significant) differences in reproductive outcomes were observed. A part of the control group consisted of women who carried germline mutations in genes other than BRCA1 or BRCA2. Control women were not suspected of diminished ovarian reserve, yet some of the genetic diseases are rare and little is known about potential reproductive implications of these mutations. Nevertheless, no statistically significant differences were observed when only controls requesting PGT because of a partner with a genetic diagnosis were included (supplementary B). Another limitation could be the use of oral contraceptives during the pre-stimulation management for timing purposes. Significantly more controls received pre-treatment with contraceptives during the ICSI cycle, in comparison to BRCA mutation carriers. Although the use of long-term hormonal contraceptives is associated with a reversible suppression of AFC and AMH serum levels [54], this association is certainly less present or even non-existing for short-term use [55]. Lastly, the carrier subgroup comprised of relatively young, healthy and normo-ovulatory BRCA mutation carriers, which could have potentially caused bias. This type of bias applies to most studies that evaluate reproductive potential in female BRCA mutation carriers, as those women are offered risk-reducing salpingo-oophorectomy (RRSO) in their mid-thirties which affects their reproduction and eligibility directly.

### Clinical Implications and Future Research

In this study, no substantial correlation between BRCA mutations and ovarian reserve conditions was observed. In conflicting studies, the size of the effect that was found was small. It remains unclear how a potential difference in AMH serum level would correlate with fecundity [56] and a difference in oocyte yield does not necessarily affect pregnancy and embryo cryopreservation rates [41]. Thus, the clinical impact of such small and ambigious differences in quantitive ovarian reserve status can be questioned and the effect of BRCA mutations on ovarian reserve may be negligible.

Future studies that evaluate qualitative aspects of ovarian reserve, such as reproductive prognosis and variables as time to pregnancy, could be of value in reproductive counselling for women with BRCA gene mutations. Future trial design could include a long-term follow-up period to assess decline patterns of AMH serum levels or other markers of ovarian reserve. Besides, the impact on age of menopause or age-related decline in oocyte quality remains to be determined in carriers of older reproductive age (i.e. those not choosing for elective oophorectomy).

## Summarising Conclusions

In conclusion, this study did not find clear differences in ovarian reserve between healthy BRCA carriers and controls. Variance in AMH serum levels can not be unambiguously addressed to carrier status, although the mentioned study limitations and limited sample size require caution. Widely varying study reports on ovarian reserve in BRCA carriers have been published and inconsistency in results might have been provoked by methodological differences. Ultimately, if there would be a difference among BRCA mutation carriers, it may not have clinically relevant implications for fertility.

## Supplementary Information

Below is the link to the electronic supplementary material.

Supplementary file1 (DOCX 14 KB)

Supplementary file2 (DOCX 35 KB)

Supplementary file3 (DOCX 37 KB)

## Data Availability

The data underlying this article cannot be shared publicly for the privacy of individuals that participated in the study. Data will be shared on reasonable request to the corresponding author.

## Abbreviations

AMH: Anti-Mullerian hormone
AFC: Antral follicle count
ICSI: Intracytoplasmic sperm injection
rFSH: Recombinant follicle stimulating hormone
PGT: Preimplantation genetic testing
GMR: Geometric mean ratio
