# Supplementary material for: The Impact of BRCA1- and BRCA2 Mutations on Ovarian Reserve Status
Source: Reprod Sci. 2022 Jun 15;30(1):270–82. doi: 10.1007/s43032-022-00997-w (PMC9810575; doi:10.1007/s43032-022-00997-w)
Supplement: Supplementary file 1 — Supplementary file1 (DOCX 14 KB) [file 43032_2022_997_MOESM1_ESM.docx]

Supplementary A: Genetic diagnoses in female non BRCA mutation carriers

| **Genetic diagnoses in female non BRCA mutation carriers (n=73)** |
| --- |
| Huntington disease *n*=10 |
| Hemofilia *n*=5 |
| Cystic Fibrosis (CF) *n*=4 |
| Neurofibromatosis *n*=4 |
| Hereditary Motory Sensory Neuropathy (HMSN) *n*=3 |
| Myotonic dystrofia *n*=3 |
| Hereditary nonpolyposis colorectal cancer (HNPCC) / Lynch / Familial adenomatous polyposis (FAP) *n*=3 |
| Duchenne muscular dystrophy *n*=2 |
| Becker muscular dystrophy *n*=2 |
| Smith lemli opitz syndrome *n*=2 |
| Other (*n*=35): Joubert syndrome, Hurler syndrome, Marfan syndrome, Kennedy’s disease, Tuberous sclerosis complex (TSC), Autosomal dominant polycystic kidney disease (ADPKD), Branchiootonorenal (BOR) syndrome, BRAT1 gene mutation, CANT1 gene mutation, congenital myopathy, congenital deafness, congenital hyperinsulinism, Familial atypical multiple mole melanoma syndrome (FAMMM), Gorlin syndrome, Distal myopathy, Li Fraumeni syndrome, OPA1 gene mutation, Leber Heredirairy Optic Neoropathy (LHON), Pyririasis rubra pilaris, Rendu Osler Weber syndrome, Seathre-Chotzen syndrome, SCN4a gene mutation, Schinzel Giedion Syndrome (SGS), SH3BP gene mutation, Spinal muscular atrophy (SMA1), Hereditary Spastic Paraplegia (HSP), SPATA5a gene mutation, TGM1 gene mutation, Oculofaciocardiodental (OFCD) syndrome, Zellweger syndrome. |

**Article**

The impact of BRCA1- and BRCA2 mutations on ovarian reserve status.
