# Supplementary material for: The Impact of BRCA1- and BRCA2 Mutations on Ovarian Reserve Status
Source: Reprod Sci. 2022 Jun 15;30(1):270–82. doi: 10.1007/s43032-022-00997-w (PMC9810575; doi:10.1007/s43032-022-00997-w)
Supplement: Supplementary file 2 — Supplementary file2 (DOCX 35 KB) [file 43032_2022_997_MOESM2_ESM.docx]

Supplementary B: Study results, excluding female non-BRCA carriers in control-group

| **Baseline Characteristics** | **BRCA mutation carrier (n=36)** | **Sig.*** | **BRCA1 mutation carrier (n=18)** | **Sig.*** | **BRCA2 mutation carrier (n=18)** | **Sig.*** | **Control (n=53)** |
| --- | --- | --- | --- | --- | --- | --- | --- |
| Female age (years) | 30.4 (±2.8) | 0.71 | 31.0 (±2.9) | 0.28 | 29.7 (±2.6) | 0.79 | 30.2 (±4.2) |
| Female BMI (kg/m^2^) | 22.7 (±3.4) | 0.27 | 23.1 (±4.3) | 0.68 | 22.4 (±2.2) | 0.58 | 23.3 (±3.7) |
| Caucasian | 36 (100%) | 0.27 | 18 (100%) | 1.000 | 18 (100%) | 1.000 | 51 (96.2%) |
| Smoking | 2 (5.6%) | 0.70 | 0 (0%) | 0.57 | 2 (11.1%) | 0.64 | 4 (7.5%) |
| Alcohol | 18 (50%) | 0.15 | 9 (50%) | 0.44 | 9 (50%) | 0.44 | 32 (60.4%) |
| Drugs | 1 (2.8%) | 0.41 | 0 (0%) | NA | 1 (5.6%) | 0.25 | 0 (0%) |
| Cycle information |  |  |  |  |  |  |  |
| Age at menarche (years) | 13.3 (±1.4) | 0.77 | 13.5 (±1.4) | 0.16 | 13.0 (±1.4) | 0.86 | 13.0 (±1.5) |
| Mean length of menstrual cycle (days) | 28.0 (±1.3) | 0.69 | 28.2 (±1.1) | 0.82 | 27.7 (±1.3) | 0.41 | 28.3 (±1.6) |
| Reproductive history |  |  |  |  |  |  |  |
| Subfertility | 3 (8.3%) | 1.000 | 2 (11.1%) | 0.64 | 1 (5.6%) | 1.000 | 4 (7.5%) |
| Prior fertility treatment | 2 (5.6%) | 0.57 | 2 (11.1%) | 0.16 | 0 (0%) | 1.000 | 1 (1.9%) |
| Nulliparity | 30 (83.3%) | 0.92 | 16 (88.9%) | 0.72 | 14 (77.8%) | 0.74 | 43 (81.1%) |
| Nulligravidity | 29 (80.6%) | 0.86 | 16 (88.9%) | 0.32 | 13 (72.2%) | 0.76 | 40 (75.5%) |
| Live birth | 5 (13.9%) | 1.000 | 2 (11.1%) | 1.000 | 3 (16.7%) | 1.000 | 8 (15.1%) |
| Family history |  |  |  |  |  |  |  |
| Early menopause (age <40 years) | 1 (2.8%) | 0.41 | 0 (0%) | NA | 1 (5.6%) | 0.25 | 0 (0%) |
| Subfertility^a^ | 3 (8.3%) | 0.166 | 1 (5.6%) | 0.45 | 2 (11.1%) | 0.16 | 1 (1.9%) |
| Menopausal age mother (years) | 49.1 (±5.8) | 0.46 | 51.5 (±6.3) | 0.64 | 47.9 (±5.2) | 0.16 | 50.3 (±5.7) |
| Breast or ovarian cancer | 33 (91.7%) | **<0.001** | 16 (88.9%) | **<0.001** | 17 (94.4%) | **<0.001** | 5 (9.4%) |
| ***AMH*** | **BRCA mutation carrier**  (n=36) | **Sig.*** | **BRCA1 mutation carrier (n=18)** | **Sig.*** | **BRCA2 mutation carrier (n=18)** | **Sig.*** | **Control (n=53)** |
| Unadjusted serum level AMH (ng/ml)  median (IQR) | 2,40 (1,80-3,00) | 0.59 | 2.40 (1.73-2.85) | 0.34 | 2.45 (1.78-3.23) | 0.12 | 1.80 (1.15 – 3.30) |
| Linear regression model |  |  |  |  |  |  |  |
| Geometric Mean Ratio (95%CI) |  |  |  |  |  |  |  |
| - BRCA carrier status, unadjusted | 1.09 (0.96-1.23) | 0.17 | 1.05 (0.36-1.23) | 0.51 | 1.13 (0.96-1.31) | 0.13 |  |
| - BRCA carrier status, age-adjusted | 1,10 (0,98-1,23) | 0.12 | 1.08 (0.93-1.25) | 0.32 | 1.11 (0.96-1.29) | 0.16 |  |
| - BRCA carrier status, fully-adjusted^c^ | 1.07 (0.96-1.21) | 0,23 | 1.04 (0.90-1.21) | 0.60 | 1.11 (0.96-1.30) | 0.16 |  |
| ***AFC*** | **BRCA mutation carrier** (n=30) | **Sig.*** | **BRCA1 mutation carrier (n=15)** | **Sig.*** | **BRCA2 mutation carrier (n=15)** | **Sig.*** | **Control (n=52)** |
| Unadjusted AFC  median (IQR) | 15.0 (10.8-20.3) | 0.53 | 14.0 (10.0-20.0) | 0.97 | 15.0 (12.0-28.0) | 0.24 | 14.0 (9.0-21.5) |
| Linear regression model |  |  |  |  |  |  |  |
| Coefficient^b^, β (95%CI) |  |  |  |  |  |  |  |
| - BRCA carrier status, unadjusted | 0.21 (-0.28-0.71) | 0.40 | -0.05 (-0.69-0.59) | 0.87 | 0.48 (-0.16-1.12) | 0.14 |  |
| - BRCA carrier status, age-adjusted | 0.22 (-0.26-0.71) | 0.37 | -0.03 (-0.66-0.60) | 0.92 | 0.48 (-0.15-1.10) | 0.13 |  |
| - BRCA carrier status, fully-adjusted^c^ | 0,22 (-0.29-0.72) | 0.40 | -0.03 (-0.69-0.63) | 0.93 | 0.46 (-0.20-1.12) | 0.17 |  |
| **ICSI/PGT cycle** | **BRCA mutation carrier (n=31 )** | ***Sig.**** | **BRCA1 mutation carrier (n=17)** | **Sig.*** | **BRCA2 mutation carrier (n=14)** | **Sig.*** | **Control (n=45)** |
| ***Received treatment*** |  |  |  |  |  |  |  |
| Long-agonist with oral contraceptive | 24 (77,4%) | **0.03** | 14 (82.4%) | 0.12 | 10 (71.4%) | **0.02** | 43 (95.6%) |
| Stimulating medicine |  | 0.15 |  | 0.72 |  | 0.08 |  |
| - rFSH | 21 (67.7%) |  | 13 (76.5%) |  | 8 (57.1%) |  | 37 (82.2%) |
| - uFSH | 10 (32.3%) |  | 4 (23.5%) |  | 6 (42.9%) |  | 8 (17.8%) |
| Start dose FSH |  |  |  |  |  |  |  |
| - 150/day | 22 (71%) | 0.43 | 12 (70.6%) | 0.54 | 10 (71.4%) | 0.75 | 28 (62.2%) |
| - 187.5/day | 1 (3.2%) | 1.000 | 0 (0%) | 1.000 | 1 (7.1%) | 0.42 | 1 (2.2%) |
| - 200/day | 0 (0%) | NA | 0 (0%) | NA | 0 (0%) | NA | 0 (0%) |
| - 225/day | 8 (25.8%) | 0.37 | 5 (29.4%) | 0.65 | 3 (21.4%) | 0.51 | 16 (35.6%) |
| Dose adjustments (FSH) |  |  |  |  |  |  |  |
| - Dose increased | 6 (19.4%) | 0.18 | 4 (23.5%) | 0.082 | 2 (14.3%) | 0.58 | 3 (6.7%) |
| - Dose decreased | 0 (0%) |  | 0 (0%) | NA | 0 (0%) | NA | 0 (0%) |
| Received cumulative dose FSH | 1925 (±543) | 0.94 | 2122 (±778) | 0.57 | 1769 (±270) | 0.56 | 1963 (±597) |
| Number of days stimulated | 11.3 (±2) | 0.45 | 11.5 (±2.8) | 0.48 | 11.1 (±1.9) | 0.63 | 10.9 (±2.2) |
| Hyper response | 5 (16.1%) | 0.75 | 1 (5.9%) | 0.66 | 4 (28.6%) | 0.23 | 6 (13.3%) |
| - Cancelled follicular punction | 1 (3.2%) |  | 0 (0%) |  | 1 (7.1%) |  | 0 (0%) |
| - >15 oocytes at follicle aspiration | 4 (12.9%) |  | 1 (5.9%) |  | 3 (21.4%) |  | 6 (13.3%) |
| ***Low response*** |  |  |  |  |  |  |  |
| Low response | 7 (22.6%) | 0.44 | 4 (23.5%) | 0.48 | 3 (21.4%) | 0.69 | 7 (15.6%) |
| Unexpected low response^d^ | 5 (71.4%) |  | 3 (75.0%) |  | 2 (66.7%) |  | 3 (42.9%) |
| - Cancelled oocyte retrieval | 5 (16.1%) |  | 3 (17.6%) |  | 2 (14.3%) |  | 4 (8.9%) |
| - <4 oocytes at retrieval | 2 (6.5%) |  | 1 (5.9%) |  | 1 (7.1%) |  | 3 (6.7%) |
| Odds-ratio (95%CI) for low response |  |  |  |  |  |  |  |
| - unadjusted | 1.58 (0.49-5.08) | 0.44 | 1.67 (0.42-6.64) | 0.47 | 1.48 (0.33-6.70) | 0.61 |  |
| - age-adjusted | 1.65 (0.49-5.54) | 0.42 | 1.51 (0.37-6.26) | 0.57 | 1.87 (0.38-9.15) | 0.44 |  |
| - fully-adjusted^e^ | 2.75 (0.58-12.93) | 0.20 | 2.82 (0.46-17.55) | 0.32 | 2.70 (0.38-19.27) | 0.13 |  |
| ***Oocyte retrieval performed*** | **BRCA mutation carrier (n=25) 80.6%** | **Sig.*** | **BRCA1 mutation carrier (n=14)**  **82.4%** | **Sig.*** | **BRCA2 mutation carrier (n=11)**  **78.6%** | **Sig.*** | **Control (n=41)**  **91.1%** |
| Total oocytes | 80.6% | 0.87 | 9 (6-12) | 0.80 | 9 (4-16) | 0.68 | 10 (5-13) |
| Mature (MII) oocytes | 7 (5-10) | 0.93 | 7 (5-10) | 0.88 | 7 (4-10) | 0.71 | 7 (5-11) |
| Fraction biopsied^f^ | 0.59 (±0.24) | 0.47 | 0.56 (±0.20) | 0.32 | 0.63 (±0.29) | 0.95 | 0.63 (±0.24) |
| Mancova, Wilk’s Lambda sig.^g^ |  |  |  |  |  |  |  |
| - unadjusted |  | 0.81 |  | 0.64 |  | 0.99 |  |
| - age-adjusted |  | 0.78 |  | 0.63 |  | 1.000 |  |
| - fully-adjusted^e^ |  | 0.73 |  | 0.83 |  | 0.82 |  |
| ***Pregnancy per started cycle*** | **BRCA mutation carrier (n=31 )** | **Sig.*** | **BRCA1 mutation carrier (n=17)** | **Sig.*** | **BRCA2 mutation carrier (n=14)** | **Sig.*** | **Control (n=45)** |
| Cycles with embryo transfer (SET) | 22 (71.0%) | 0.99 | 12 (70.6%) | 1.000 | 10 (71.4%) | 1.000 | 32 (71.1%) |
| Pregnancy | 8 (25,6%) | 0.72 | 4 (23.5%) | 1.000 | 4 (28.6%) | 0.72 | 10 (22.2%) |
| - biochemical pregnancy, miscarriage or molar pregnancy | 4 (12.9%) |  | 2 (11.8%) |  | 2 (14.3%) |  | 3 (6.6%) |
| - ongoing clinical pregnancy^h^ | 4 (12.9%) |  | 2 (11.8%) |  | 2 (14.3%) |  | 7 (15.6%) |
| Odds-ratio (95%CI) for ongoing pregnancy |  |  |  |  |  |  |  |
| - unadjusted | 0.91 (0.27-3.03) | 0.88 | 0.73 (0.14-3.89) | 0.71 | 0.91 (0.17-4.95) | 0.91 |  |
| - age-adjusted | 0.91 (0.27-3.03) | 0.87 | 0.65 (0.12-3.55) | 0.62 | 1.01 (0.18-5.72) | 0.99 |  |
| - fully-adjusted^e^ | 1.16 (0.29-4.68) | 0.84 | 0.98 (0.14-6.90) | 0.14 | 0.66 (0.06-8.00) | 0.75 |  |

*BMI, body-mass index; PGT, pre-implantation genetic testing; EUG, extra uterine gravidity; AMH, anti-Mullerian hormone; AFC, antral follicle count; IQR, interquartile range; rFSH/uFSH, recombinant/urinary follicle-stimulating hormone; SET, single embryo transfer; no, number; SD, standard deviation; CI, confidence interval*

Values presented as number (%) in categorical variables, values presented as mean (SD) or median (IQR) in continuous variables.

Median AFC (2-10mm) in both ovaries assessed using a standard transvaginal sonography (TVS)

* P-values calculated using Fisher’s exact/Chi-square or Mann-Whitney U/T-test

^a^ Subfertility issues in mother, sister and/or aunt (mothers side) necessitating referral for fertility investigation or treatment.

^b^ Estimated coefficient (β) of BRCA carrier status on the natural logarithm of AMH serum levels, retransformed into the original scale (Exp(β)) or estimated coefficient of BRCA carrier status on square-root transformed AFC

^c^ Adjusted for age, BMI, gravidity, smoking and oral contraceptive use in downregulation

^d^ Low response was expected in females with AMH<0.96 ng/ml or AFC 0-7

^e^ Adjusted for age, BMI, gravidity, smoking, oral contraceptive use in downregulation, type- and cumulative dosage of administered gonadotropin
^f^ Number of embryos that was biopsied, divided by the number of retrieved oocytes
^g^ Level of significance for variance in total oocytes, mature oocytes or fraction biopsied, explained by BRCA carrier status
^h^ ongoing clinical pregnancy with fetal heartbeat at 7 weeks of gestation

**Article**

The impact of BRCA1- and BRCA2 mutations on ovarian reserve status.
