# Supplementary material for: The Impact of BRCA1- and BRCA2 Mutations on Ovarian Reserve Status
Source: Reprod Sci. 2022 Jun 15;30(1):270–82. doi: 10.1007/s43032-022-00997-w (PMC9810575; doi:10.1007/s43032-022-00997-w)
Supplement: Supplementary file 3 — Supplementary file3 (DOCX 37 KB) [file 43032_2022_997_MOESM3_ESM.docx]

Supplementary C: Study results of all subjects included in full analyses

| **Baseline Characteristics** | **BRCA mutation carrier (n=42)** | ***Sig.**** | **BRCA1 mutation carrier (n=22)** | ***Sig.**** | **BRCA2 mutation carrier (n=20)** | ***Sig.**** | **Control (n=135)** |
| --- | --- | --- | --- | --- | --- | --- | --- |
| Female age (years) | 30.5 (±3.0) | 0.85 | 30.7 (±3.1) | 0.63 | 30.3 (±3.1) | 0.83 | 30.5 (±4.0) |
| Female BMI (kg/m^2^) | 22.6 (±3.3) | 0.17 | 22.9 (±4.0) | 0.43 | 22.2 (±2.2) | 0.19 | 23.7 (±4,1) |
| Caucasian | 42 (100%) | 0.12 | 22 (100%) | 0.36 | 20 (100%) | 0.61 | 126 (93.3%) |
| Smoking | 2 (4.8%) | 1.000 | 0 (0%) | 0.36 | 2 (10%) | 0.64 | 9 (6.7%) |
| Alcohol | 19 (45.2%) | 0.56 | 10 (45.5%) | 0.67 | 9 (45%) | 0.65 | 68 (50.4%) |
| Drugs | 1 (2.4%) | 0.42 | 0 (0%) | 1.000 | 1 (5%) | 0.24 | 1 (0.7%) |
| PGT indication |  |  |  |  |  |  |  |
| - Female BRCA1 mutation carrier | 22 (52.4%) | NA | 22 (100%) | NA | NA | NA | N/A |
| - Female BRCA2 mutation carrier | 20 (47.6%) |  | NA |  | 20 (100%) |  | N/A |
| - Female non BRCA mutation carrier | N/A |  | NA |  | NA |  | 79 (58.5%) |
| - Male non BRCA mutation carrier | N/A |  | NA |  | NA |  | 56 (41.5%) |
| Cycle information |  |  |  |  |  |  |  |
| Age at menarche (years) | 13.2 (±1.4) | 0.24 | 13.5 (±1.4) | 0.11 | 13.0 (±1.4) | 0.90 | 13.0 (±1.4) |
| Mean length of menstrual cycle (days) | 28.1 (±1.7) | 0.37 | 28.6 (±1.7) | 0.82 | 27.5 (±1.4) | 0.11 | 28.4 (±1.9) |
| Reproductive history |  |  |  |  |  |  |  |
| Subfertility | 3 (7.1%) | 0.72 | 2 (9.1%) | 0.63 | 1 (5%) | 1.000 | 8 (5.9%) |
| Prior fertility treatment | 2 (4.8%) | 0.24 | 2 (9.1%) | 0.10 | 0 (0%) | 1.000 | 2 (1.5%) |
| Nulliparity | 33 (78.6%) | 0.23 | 17 (77.3%) | 0.43 | 16 (80%) | 0.31 | 93 (68.9%) |
| Nulligravidity | 32 (76.2%) | 0.16 | 17 (77.3%) | 0.24 | 15 (75%) | 0.35 | 87 (64.4%) |
| Live birth | 6 (14.3%) | 0.10 | 3 (13.6%) | 0.19 | 3 (15%) | 0.26 | 36 (26.7%) |
| Family history |  |  |  |  |  |  |  |
| Early menopause (age <40 years) | 1 (2.4%) | 0.24 | 0 (0%) | NA | 1 (5%) | 0.13 | 0 (0%) |
| Subfertility^a^ | 4 (9.5%) | 0.25 | 1 (4.5%) | 1.000 | 3 (15%) | 0.09 | 6 (4.4%) |
| Menopausal age mother (years) | 49.2 (±5.8) | 0.43 | 51.8 (±5.5) | 0.35 | 47.4 (±5.4) | 0.07 | 50.6 (±4.4) |
| Breast or ovarian cancer | 39 (92.9%) | **<0.001** | 20 (90.9%) | **<0.001** | 19 (95%) | **<0.001** | 7 (5.2%) |
| ***AMH*** | **BRCA mutation carrier (n=42)** | ***Sig.**** | **BRCA1 mutation carrier (n=22)** | **Sig.*** | **BRCA2 mutation carrier (n=20)** | **Sig.*** | **Control (n=135)** |
| Unadjusted serum level AMH (ng/ml)  median (IQR) | 2,35 (1,5-2,85) | 0,95 | 2,35 (1,4-2,8) | 0,64* | 2,35 (1,55-3,15) | 0,69* | 2,00 (1,20-3,30) |
| Linear regression model |  |  |  |  |  |  |  |
| Geometric Mean Ratio (95%CI) |  |  |  |  |  |  |  |
| - BRCA carrier status, unadjusted | 0,98 (0,88-1,09) | 0,68 | 0,94 (0,82-1,09) | 0,44 | 1,01 (0,87-1,18) | 0,86 |  |
| - BRCA carrier status, age-adjusted | 0,98 (0,89-1,09) | 0,66 | 0,95 (0,83-1,09) | 0,46 | 1,01 (0,87-1,17) | 0,92 |  |
| - BRCA carrier status, fully-adjusted^c^ | 0,97 (0,87-1,09) | 0,62 | 0,93 (0,81-1,08) | 0,34 | 1,02 (0,88-1,18) | 0,82 |  |
| ***AFC*** | **BRCA mutation carrier (n=38)** | ***Sig.**** | **BRCA1 mutation carrier (n=20)** | **Sig.*** | **BRCA2 mutation carrier (n=18)** | **Sig.*** | **Control (n=130)** |
| Unadjusted AFC  median (IQR) | 13.5 (9.0-18.5) | 0.69 | 12.0 (9.0-17.5) | 0.33 | 15.0 (9.5-22.0) | 0.68 | 14.0 (9.0-20,0) |
| Linear regression model |  |  |  |  |  |  |  |
| Coefficient^b^, β (95%CI) |  |  |  |  |  |  |  |
| - BRCA carrier status, unadjusted | -0.03 (-0.42-0.36) | 0.88 | -0.27 (-0.77-0.24) | 0.30 | 0.23 (-0.30-0.76) | 0.39 |  |
| - BRCA carrier status, age-adjusted | -0,05 (-0.43-0.34) | 0.82 | -0.28 (-0.78-0.23) | 0.28 | 0.21 (-0.31-0.74) | 0.43 |  |
| - BRCA carrier status, fully-adjusted^c^ | -0.003 (-0.42-0.41) | 1.00 | -0.25 (-0.78-0.28) | 0.35 | 0.27 (-0.28-0.83) | 0.34 |  |
| **ICSI/PGT cycle** | **BRCA mutation carrier (n=42 )** | ***Sig.**** | **BRCA1 mutation carrier (n=22)** | **Sig.*** | **BRCA2 mutation carrier (n=20)** | **Sig.*** | **Control (n=135)** |
| ***Received treatment*** |  |  |  |  |  |  |  |
| Long-agonist with oral contraceptive | 28 (66.7%) | **<0.001** | 15 (68.2%) | **0.01** | 13 (65%) | **<0.001** | 123 (91.1%) |
| Stimulating medicine |  | **<0.001** |  | **0.02** |  | **<0.001** |  |
| - rFSH | 27 (64.3%) |  | 15 (68.2%) |  | 12 (60%) |  | 119 (88.1%) |
| - uFSH | 15 (35.7%) |  | 7 (31.8%) |  | 8 (40%) |  | 16 (11.9%) |
| Start dose FSH |  |  |  |  |  |  |  |
| - 75/day | 1 (2.4%) | 1.000 | 0 (0%) | NA | 1 (5%) | 1.000 | 0 (0%) |
| - 100/day | 1 (2.4%) | 0.12 | 1 (4.5%) | 0.70 | 0 (0%) | 0.22 | 14 (10.4%) |
| - 112.5/day | 1 (2.4%) | 0.42 | 0 (0%) | 1.000 | 1 (5%) | 0.24 | 1 (0.7%) |
| - 125/day | 2 (4.8%) | 0.63 | 0 (0%) | 1.000 | 2 (10%) | 0.17 | 4 (3.0%) |
| - 150/day | 25 (59.5%) | 0.59 | 14 (63.6%) | 0.44 | 11 (55%) | 0.99 | 74 (54.8%) |
| - 187.5/day | 1 (2.4%) | 1.000 | 0 (0%) | 1.000 | 1 (5%) | 0.43 | 3 (2.2%) |
| - 200/day | 0 (0%) | 1.000 | 0 (0%) | 1.000 | 0 (0%) | 1.000 | 1 (0.7%) |
| - 225/day | 11 (26.2%) | 0.80 | 7 (31.8%) | 0.72 | 4 (20%) | 0.44 | 38 (28.1%) |
| Dose adjustments (FSH) |  |  |  |  |  |  |  |
| - Dose increased | 8 (19.0%) | 0.04 | 5 (22.7%) | **0.04** | 3 (15%) | 0.38 | 10 (7.4%) |
| - Dose decreased | 0 (0%) | N/A | 0 (0%) | NA | 0 (0%) | NA | 0 (0%) |
| Received cumulative dose FSH | 1871 (±629) | 0.84 | 2094 (±739) | 0.26 | 1699 (±579) | 0.33 | 1895 (±647) |
| Number of days stimulated | 11.3 (±2.5) | 0.99 | 11.6 (±2.5) | 0.43 | 10.9 (±2.4) | 0.65 | 11.2 (±2.6) |
| Hyper response | 8 (19%) | 0.93 | 1 (4.5%) | **0.03** | 7 (35%) | 0.35 | 34 (25,2%) |
| - Cancelled follicular punction | 2 (4.8%) |  | 0 (0%) |  | 2 (10%) |  | 6 (4.4%) |
| - >15 oocytes at follicle aspiration | 6 (14.3%) |  | 1 (4.5%) |  | 5 (25%) |  | 28 (20.7%) |
| ***Low response*** |  |  |  |  |  |  |  |
| Low response | 9 (21.4%) | 0.06 | 5 (22.7%) | 0.15 | 4 (20%) | 0.26 | 14 (10.4%) |
| Unexpected low response^d^ | 5 (55.6%) |  | 3 (60%) |  | 2 (50%) |  | 6 (42.8%) |
| - Cancelled oocyte retrieval | 6 (14.3%) |  | 3 (13.6%) |  | 3 (15%) |  | 9 (6.7%) |
| - <4 oocytes at retrieval | 3 (7.1%) |  | 2 (9.1%) |  | 1 (5%) |  | 5 (3.7%) |
| Odds-ratio (95%CI) for low response |  |  |  |  |  |  |  |
| - unadjusted | 2.36 (0.94-5.92) | 0.07 | 2.54 (0.81-7.95) | 0.11 | 2.16 (0.63-7.37) | 0.22 |  |
| - age-adjusted | 2.76 (1.05-7.30) | **0.04** | 2.88 (0.88-9.44) | 0.08 | 2.63 (0.73-9.51) | 0.14 |  |
| - fully-adjusted^e^ | 3.17 (0.87-11.49) | 0.08 | 2.75 (0.70-11.27) | 0.16 | 3.43 (0.75-15.71) | 0.11 |  |
| ***Oocyte retrieval performed*** | **BRCA mutation carrier (n=34),**  **81.0%** | ***Sig.**** | **BRCA1 mutation carrier (n=19)**  **86.4%** | **Sig.*** | **BRCA2 mutation carrier (n=15)**  **75%** | **Sig.*** | **Control**  **(n=119)**  **88.1%** |
| Total oocytes | 9 (5-14) | 0.13 | 9 (6-11) | 0.09 | 9 (5-16) | 0.77 | 11 (8-15) |
| Mature (MII) oocytes | 7 (4-7) | 0.13 | 7 (4-10) | 0.17 | 7 (4-13) | 0.70 | 9 (6-12) |
| Fraction biopsied^b^f | 0.30 (±0.22) | 0.59 | 0.57 (±0.18) | 0.30 | 0.64 (±0.26) | 0.96 | 0.63 (±0.24) |
| Mancova, Wilk’s Lambda sig.^g^ |  |  |  |  |  |  |  |
| - unadjusted |  | 0.40 |  | 0.23 |  | 0.94 |  |
| - age-adjusted |  | 0.39 |  | 0.23 |  | 0.93 |  |
| - fully-adjusted^e^ |  | 0.82 |  | 0.61 |  | 0.87 |  |
| ***Pregnancy per started cycle*** | **BRCA mutation carrier (n=42)** | ***Sig.**** | **BRCA1 mutation carrier (n=22)** | **Sig.*** | **BRCA2 mutation carrier (n=20)** | **Sig.*** | **Control (n=135)** |
| Cycles with embryo transfer (SET) | 29 (69.0%) | 0.73 | 15 (68.2%) | 0.72 | 14 (70%) | 0.86 | 97 (71.9%) |
| Pregnancy | 12 (28.6%) | 0.52 | 6 (27.3%) | 0.72 | 6 (30%) | 0.58 | 32 (23.7%) |
| - biochemical pregnancy, miscarriage or molar pregnancy | 6 (14.3%) |  | 2 (8.0%) |  | 4 (20%) |  | 10 (7.4%) |
| - ongoing clinical pregnancy^h^ | 6 (14.3%) |  | 4 (18.2%) |  | 2 (10%) |  | 22 (16.3%) |
| Odds-ratio (95%CI) for ongoing pregnancy |  |  |  |  |  |  |  |
| - unadjusted | 0.86 (0.32-2.28) | 0.76 | 1.14 (0.35-3.70) | 0.83 | 0.57 (0.12-2.64) | 0.47 |  |
| - age-adjusted | 0.86 (0.32-2.28) | 0.76 | 1.14 (0.35-3.69) | 0.83 | 0.57 (0.12-2.65) | 0.48 |  |
| - fully-adjusted^e^ | 1.23 (0.38-3.95) | 0.74 | 2.03 (0.54-7.65) | 0.30 | 0.45 (0.05-3.83) | 0.46 |  |

*BMI, body-mass index; PGT, pre-implantation genetic testing; EUG, extra uterine gravidity; AMH, anti-Mullerian hormone; AFC, antral follicle count; IQR, interquartile range; rFSH/uFSH, recombinant/urinary follicle-stimulating hormone; SET, single embryo transfer; no, number; SD, standard deviation; CI, confidence interval*

Values presented as number (%) in categorical variables, values presented as mean (SD) or median (IQR) in continuous variables.

Median AFC (2-10mm) in both ovaries assessed using a standard transvaginal sonography (TVS)

* P-values calculated using Fisher’s exact/Chi-square or Mann-Whitney U/T-test

^a^ Subfertility issues in mother, sister and/or aunt (mothers side) necessitating referral for fertility investigation or treatment.

^b^ Estimated coefficient (β) of BRCA carrier status on the natural logarithm of AMH serum levels, retransformed into the original scale (Exp(β)) or estimated coefficient of BRCA carrier status on square-root transformed AFC

^c^ Adjusted for age, BMI, gravidity, smoking and oral contraceptive use in downregulation

^d^ Low response was expected in females with AMH<0.96 ng/ml or AFC 0-7

^e^ Adjusted for age, BMI, gravidity, smoking, oral contraceptive use in downregulation, type- and cumulative dosage of administered gonadotropin
^f^ Number of embryos that was biopsied, divided by the number of retrieved oocytes
^g^ Level of significance for variance in total oocytes, mature oocytes or fraction biopsied, explained by BRCA carrier status
^h^ ongoing clinical pregnancy with fetal heartbeat at 7 weeks of gestation

**Article**

The impact of BRCA1- and BRCA2 mutations on ovarian reserve status.
